# *In vitro* Effect of Photodynamic Therapy with Curcumin and Methylene Blue Photosensitizers on Staphylococcus Aureus

**DOI:** 10.30476/DENTJODS.2021.90146.1470

**Published:** 2022-09

**Authors:** Sarvin Entezari, Nahid Moezzimoghadam, Shirin Lawaf, Arash Azizi

**Affiliations:** 1 Dentist, Faculty of Dentistry, Tehran Medical Sciences. Islamic Azad University, Tehran, Iran; 2 Dept. of Oral Medicine, Faculty of Dentistry, Tehran Medical Sciences. Islamic Azad University, Tehran, Iran; 3 Dept. of Prosthodontics, Faculty of Dentistry, Tehran Medical Sciences, Islamic Azad University, Tehran, Iran

**Keywords:** Staphylococcus Aureus, Photodynamic Therapy, Photochemotherapy, Photosensitizing agent, Photosensitivity disorders, Curcumin, Methylene Blue

## Abstract

**Statement of the Problem:**

Staphylococcus aureus (S.A) can colonize in the skin, nasal cavity, and oral cavity. In the oral cavity, it can cause dental caries and periodontal disease. Mouthwashes can be used as an adjunct to mechanical plaque control methods to decrease the load of oral microorganisms. Chlorhexidine (CHX) is a commonly used antimicrobial mouthwash with side effects such as changing the sense of taste, tooth discoloration, oral mucosal burning, allergy, and xerostomia. It also has adverse systemic effects, if swallowed.

**Purpose:**

This study aimed to assess the effect of photodynamic therapy (PDT) with curcumin and methylene blue (MB) photosensitizers and different laser parameters on S.A colony count.

**Materials and Method:**

In this in vitro experimental study, 99 samples of standard-strain S.A were subjected to PDT with curcumin and MB photosensitizers with/without irradiation of 660 and 445 nm laser with different exposure parameters, and CHX in 9 groups (n=11). The samples were cultured in microplates containing Mueller-Hinton agar, and the number of colony forming units (CFUs) was counted after 24 h of incubation at 37°C. Data were analyzed by the Kruskal-Wallis and Dunn tests.

**Results:**

The minimum colony count was noted in CHX group (CFUs=0) followed by MB and 660nm diode laser group irradiated for 100 s (CFUs=147.2727±169.35707). The difference in this respect was significant between MB+660nm diode laser for 100 s and other groups (*p*< 0.05) except for the MB + 660 nm diode laser for 60 s group.

**Conclusion:**

CHX is superior to laser for elimination of S.A. However, PDT with 660 nm diode laser + MB has considerable antimicrobial efficacy against S.A; increasing the duration of laser irradiation enhances the antimicrobial effect.

## Introduction

Staphylococcus aureus (*S.A*) colonizes the skin, nasal cavity, and oral cavity, and is the most important human pathogen [ 1 ]. It has been isolated from the oral cavity, and is associated with bacterial infection of the salivary glands (particularly the parotid gland), angular cheilitis, denture stomatitis, and acute dentoalveolar infection. It is a part of the oral microflora [ 2 - 3 ] and can colonize in the oral cavity and subsequently cause dental caries and periodontal disease [ 4 ]. 

Mouthwashes are commonly used as an adjunct to mechanical plaque control methods to further decreasing the count of oral microorganisms [ 5 ]. Chlorhexidine (CHX) is a commonly used antibacterial mouthwash with a broad-spectrum antibacterial activity. However, it has drawbacks such as altering the sense of taste, discoloration of tooth and restoration surfaces, oral mucosal burning, allergy, xerostomia, and adverse systemic effects, if swallowed. These side effects limit the application of CHX [ 6 ]. Photodynamic therapy (PDT) requires three components of light, photosensitizer, and free radicals. In PDT, a certain wavelength of light is used to activate the photosensitizer and generate free radicals to eliminate the target cells. In dentistry, this modality is used to prevent the proliferation of microorganisms responsible for dental caries and periodontitis. Some of benefits of PDT include being non-invasiveness, not requiring antibiotics, and the potential of destruction of bacteria in a short period of time [ 7 ]. Evidence shows that curcumin and MB can inhibit bacterial proliferation when irradiated with a specific wavelength of light [ 8 ]. 

Recently, PDT has gained increasing popularity for elimination of microorganisms [ 9 ]. Many of the bacteria and fungi that are part of the oral microflora have shown sensitivity to PDT [ 10 - 12 ]. Azizi *et al.* [ 13 - 15 ] evaluated the effects of PDT with methylene blue (MB) on *Streptococcus mutans* ( *S.M*), *Lactobacillus acidophilus* ( *L.A*), and *Candida albicans* ( *C.A*) and reported a significant reduction in the count of all tested microorganisms following PDT. In all these studies, PDT presented good effects on microorganisms and decreased CFU microorganisms [ 13 - 15 ]. Recently, blue lasers were introduced to the market with several applications as in oral soft tissue surgery [ 16 ]. Blue lasers can be used as the light source in PDT against *S.M*, *Enterococcus faecalis*, and C.A, with curcumin photosensitizer [ 16 ]. 

Considering the fact that PDT is easily available, low-cost, and non-invasive, and since the effect of PDT with blue laser and curcumin on *S.A* has not been previously evaluated, this in vitro study aimed to assess the effect of PDT with curcumin and MB on *S.A*

## Materials and Method

This in vitro experimental study was conducted at the laser Department of School of dentistry, Tehran Islamic Azad University of Medical Sciences in 2019-2020. This study was approved by Ethic Committee of Dental School of Tehran Islamic Azad University. The ethics code number was IR.IAU.DENTAL.REC.1399,57. 

### Preparation of microbial suspension with 0.5 McFarland standard concentration

*S.A* colonies were transferred into a test tube containing saline by a swab, to prepare a microbial suspension with 0.5 McFarland standard concentration, containing 1.5x108 colony forming units (CFUs)/mL. If the turbidity was lower than 0.5 McFarland standard concentration, some more colonies were added, and if the turbidity was higher than 0.5 McFarland concentration, some more sterile saline was added to reach the desired turbidity. To ensure 0.5 McFarland standard concentration, a spectrophotometer was used (it had to show a value in the range of 0.08 to 0.13 at 625 nm wavelength). 

### Preparation of Mueller Hinton agar culture medium

For this purpose, 38 g of Mueller Hinton agar powder (Sigma, Germany) was added to 1 L of water, and heated until completely dissolved. The solution was then autoclave-sterilized at 121°C for 15min and then was allowed to cool down for 1h. It was then poured into the plates. 

### Laser

**Figure 1:**
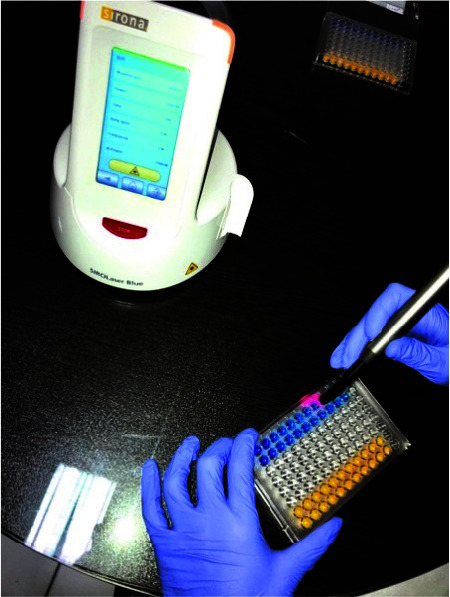
Diode laser irradiation of microplates

Diode laser (Sirona, Germany) at 445 and 660nm wavelengths was used in this study (Figure 1). The 445nm diode laser had 200mW power while the 660nm laser had 100mW power. The laser handpiece was calibrated prior to use.

### Photosensitizers

In this study, 0.02% MB (Merck, Germany) was used as photosensitizer, which is activated at 660nm wavelength. To prepare 0.02% MB, 2mg MB powder was mixed with 10 cc sterile saline. Curcumin with 10.2% concentration (Sigma, Germany) was also used as another photosensitizer. To prepare 10.2% curcumin, 1.2g curcumin powder was dissolved in 10 cc of 4% dimethyl sulfoxide. First, a standard suspension of *S.A*ureus (ATCC 25-25923) with 0.5 McFarland standard concentration (containing 1.5x108CFUs/mL) was prepared; 0.1mL of this suspension was transferred into wells of a microplate by a sterile sampler. Also, 0.1mL of the respective photosensitizer or 0.2% sterile CHX was added to each well. All phases of the experiment were performed under a laminar hood to ensure sterile and dark environment.

**Figure 2:**
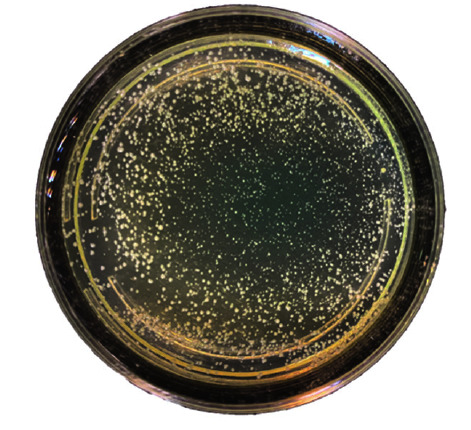
Bacterial proliferation after 660nm diode laser irradiation for 60 s plus methylene blue photosensitizer

**Figure 3:**
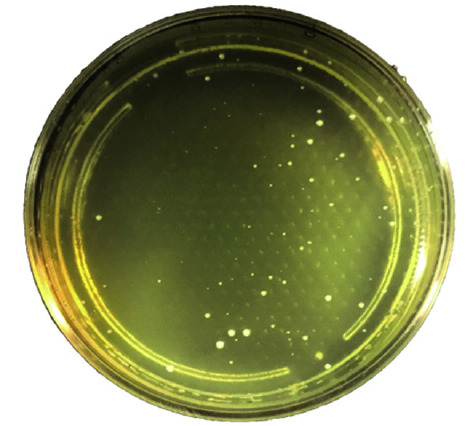
Bacterial proliferation after 660 nm diode laser irradiation for 100 s plus methylene blue photosensitizer

**Figure 4:**
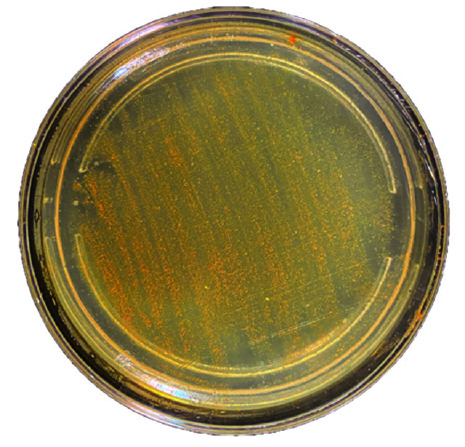
Bacterial proliferation after 445 nm blue laser irradiation for 40 s plus curcumin photosensitizer

**Figure 5:**
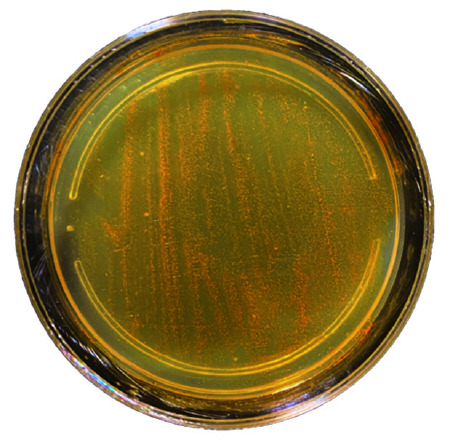
Bacterial proliferation after 445 nm blue laser irradiation for 25 s plus curcumin photosensitizer

**Figure 6:**
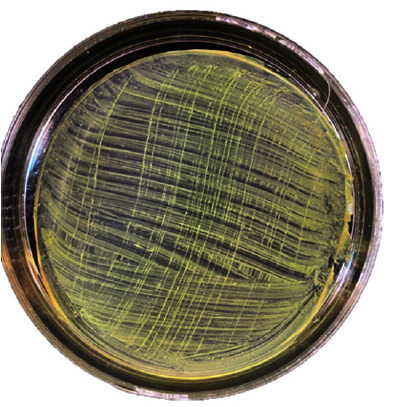
Pure culture medium with S. aureus (positive control)

Laser was irradiated to the surface of suspension form 1cm distance. After the intervention, all samples were cultured on Mueller Hinton agar and incubated at 37°C for 24h. The number of colonies was then counted and reported as CFUs/mL [ 15 - 16 ]. The samples were evaluated in 9 groups (n=11) as follows:

1. Diode laser with 660nm wavelength and 100mW power for 60s+MB photosensitizer and *S.A* (Figure 2) 

2. Diode laser with 660 nm wavelength and 100 mW power for 100 s+MB photosensitizer and *S.A* (Figure 3)

3. Blue laser with 445nm wavelength and 200mW power for 40s+curcumin photosensitizer and *S.A* (Figure 4)

4. Blue laser with 445nm wavelength and 200mW power for 25s+curcumin photosensitizer and *S.A* (Figure 5)

5. MB with *S.A* without laser irradiation

6. Curcumin with *S.A* without laser irradiation

7. Culture medium with *S.A* and CHX

8. Pure culture medium without *S.A*(negative control group)

9. Pure culture medium with *S.A* but without photosensitizer or laser (positive control group) (Figure 6)

### Statistical analysis

The SPSS version 18 was used for data analysis and level of significance was set at *p*<0.05. Since data were not normally distributed, statistical analysis was carried out using non-parametric Kruskal-Wallis and Dunn tests. 

## Results

This experimental study evaluated the effect of PDT with 660nm diode laser and 445nm blue laser with MB and curcumin photosensitizers on *S.A*. Table 1 shows the measures of central dispersion of *S.A* colony count. A total of 9 groups including 7 experimental groups and one positive and one negative control group were evaluated. The following results were obtained. The colony count was zero in the negative control and CHX groups. Among the 7 experimental groups, diode laser with 660nm wavelength for 100 s with MB photosensitizer was the most effective in elimination of *S.A* colonies (147.2727±169.35707 CFUs/mL) while the curcumin group without laser irradiation was the least effective (10409090.9091±6040770.57095 CFUs/mL).

**Table 1:** Measures of central dispersion of S. aureus colony count in different groups

Group	Mean	Std. deviation	Minimum	Maximum
660 nm diode laser for 60 s + MB	3954.5455	610.51394	3500	5000
660 nm diode laser for 100 s + MB	147.2727	169.35707	20	600
445 nm blue laser for 40 s + curcumin	59090.9091	47000.96711	104	105
445 nm blue laser for 25 s + curcumin	836363.6364	364067.92573	105	106
MB* without laser	6727272.7273	4540724.81199	106	107
Curcumin without laser	10409090.9091	6040770.57095	1.5×106	1.5×107
Culture medium with S. aureus† and CHX‡	0	0	0	0
Pure culture medium with S. aureus	1.5×108	0	1.5×108	1.5×108

* Methylene Blue

† Staphylococcus Aureus

‡ Chlorhexidine

The maximum colony count was noted in the positive control group (1.5x108CFUs/mL). The non-parametric Kruskal-Wallis test showed that each experimental group had a significant difference with the positive control group (*p*< 0.0001). Table 2 shows pairwise comparisons of the groups. As shown, diode laser with 660 nm wavelength for 100s+MB had significant differences with other groups in colony count (*p*< 0.05), except for the diode laser with 660 nm wavelength for 60s+MB group. In addition, a significant difference in colony count was noted between 660nm diode laser with MB (maximum number of CFUs) and the curcumin group (minimum number of CFUs) (*p*< 0.05).

**Table 2:** Pairwise comparisons of the groups regarding S. aureus colony count (CFU*s/mL)

Group	*p* Value
Laser, MB**, 100s***– Laser, MB, 60s	0.176
Laser, MB, 100s– Laser, MB, 40s*	0.006
Laser, MB, 100s– Laser, curcumin, 25s*	0.000
Laser, MB, 100s– MB*	0.000
Laser, MB, 100s– curcumin*	0.000
Laser, MB, 60s,– Laser, curcumin, 40s	0.155
Laser, MB, 60s– Laser, curcumin, 25s*	0.004
Laser, MB, 60s– MB*	0.000
Laser, MB, 60s– curcumin*	0.000
Laser, curcumin, 40s– Laser, curcumin, 25s	0.155
Laser, curcumin, 40s– MB*	0.006
Laser, curcumin, 40s– curcumin*	0.000
Laser, curcumin, 25s– MB	0.183
Laser, curcumin, 25s– curcumin*	0.024
MB– curcumin	0.353

* Colony Forming Unit

** Methylene Blue

*** Significant difference

According to the results, laser irradiation with 660nm and 445nm wavelengths along with MB and curcumin photosensitizers decreased the bacterial count, and its antibacterial efficacy improved with an increase in duration of laser irradiation, such that irradiation of 660nm laser for 100s with MB had maximum efficacy for elimination of *S.A*. 

## Discussion

This in vitro study evaluated the effect of PDT with Curcumin and MB plus diode laser on *S.A*. Many studies have suggested PDT for elimination of microorganisms such as *S.M* and have discussed that it can be used as an alternative to CHX mouthwash [ 13 - 16 ].

Azizi *et al.* [ 17 ] evaluated the effects of PDT with MB and curcumin on *S.M* using continuous and pulsed laser modes. They reported maximum reduction in colony count following continuous laser irradiation and use of curcumin as photosensitizer. They demonstrated that use of laser plus photosensitizer had significant antibacterial efficacy. However, CHX is still the gold-standard antimicrobial agent, despite its shortcomings and complications. Use of two modes of laser irradiation was strength of their study, and they showed that continuous laser irradiation was more effective than pulsed mode for bacterial elimination. Similar to our study, they used curcumin and MB photosensitizers; however, the main advantage of our study was evaluation of different lasers with different exposure parameters. Nemezio *et al.* [ 18 ] evaluated the efficacy of PDT with MB against *S.M*. They showed that light source or photosensitizer alone had no antimicrobial effect; however, their combination enhanced the elimination of microorganisms. They irradiated laser twice a day, and showed that PDT with MB caused a significant reduction in bacterial count, comparable to CHX group. However, we evaluated *S.A* and the results showed that CHX had maximum efficacy followed by PDT with MB and laser.

Increasing the duration of laser irradiation improved the antibacterial efficacy. Comparison of nine groups and different laser irradiation periods was strength of our study. Paschoal *et al.* [ 16 ] evaluated the efficacy of PDT with curcumin and LED blue laser against *S.M*.

They reported significant reduction of bacterial colonies in this group, compared with other groups. Their results were in line with our findings that showed that 445nm laser+ curcumin was more effective than curcumin alone for elimination of S. aureus. The main advantage of their study was the use of different doses of curcumin and LED alone and in combination with each other. However, high doses of curcumin can damage the oral mucosa, and lower concentrations of curcumin are preferred to protect the oral tissue [ 19 ]. Dovigo *et al.* [ 20 ] evaluated the effect of PDT on oral candidiasis in rats. They inoculated the oral cavity of immunocompromised rats with C.A and then performed PDT with different concentrations of topical curcumin plus LED radiation. The results were compared with a control group. They reported that PDT with curcumin and laser caused a greater reduction in C.A colony count with no adverse effect. Their results were in accordance with our findings. Their study was conducted on animal models, which was strength of their study. Also, different concentrations of curcumin plus laser were compared with the control group. We evaluated different doses of laser in different groups, which was an advantage. 

Azizi *et al.* [ 12 ] evaluated the effects of PDT with MB and indocyanine green on *L.A.* They found that use of MB alone and in combination with 660 nm laser had high inhibitory effect on *L.A.* They showed that MB had higher antibacterial efficacy than indocyanine green. Their study was somehow similar to our study since they had several groups, used laser with different doses, and CHX as the control group. However, the main advantage of our study was evaluation of different durations of laser irradiation. Also, our study showed greater antibacterial efficacy in longer irradiation of laser plus the use of photosensitizer; use of photosensitizer alone was less effective. 

Searching the literature by the authors yielded no study on the effects of PDT with different photosensitizers, in comparison with CHX control group, on *S.A*, and this study appears to be the first on this topic. 

Curcumin has various biological properties. It can inhibit the proliferation of cancer cells and serve as an anti-oxidant. In addition, it has antimicrobial activity against gram-positive and gram-negative bacteria [ 16 ]. Efficacy of a photosensitizer depends on three factors including its ability to bind to the bacterial membrane, to penetrate into the cells, and to generate free radicals around the bacteria when irradiated by light [ 16 ]. 

MB is an alkaline photosensitizer that can pass through the bacterial cell membrane, affect the bacterial genome, and eliminate the bacteria. In addition, when irradiated by laser, it generates free oxygen species that eliminate the bacteria [ 16 - 18 ].

*S.A* is a gram-positive microorganism, with a thick cell wall, which is not highly permeable and inhibits the passage of hydrophobic materials [ 18 ]. This peptidoglycan layer has selective permeability against simple diffusion, and penetration of photosensitizer molecules depends on their size and degree of solubility [ 18 ]. 

CHX is a commonly used antimicrobial agent in dentistry, which effectively decreases the bacterial viability. CHX is the gold-standard against microbial biofilm. Our study confirmed the antibacterial activity of CHX against *S.A*, since it showed maximum antibacterial activity against *S.A*. 

## Conclusion

This study showed the superiority of CHX for elimination of *S.A* compared with laser irradiation. However, use of 660 nm diode laser + MB had significant antibacterial effect on S. aureus, and increasing the laser irradiation time enhanced its antimicrobial activity.

## Conflict of Interest

The authors declare that they have no conflicts of interests.
